# PCQNet: A Trainable Feedback Scheme of Precoder for the Uplink Multi-User MIMO Systems

**DOI:** 10.3390/e24081066

**Published:** 2022-08-02

**Authors:** Xiuwen Bao, Ming Jiang, Wenhao Fang, Chunming Zhao

**Affiliations:** 1 National Mobile Communications Research Laboratory, Southeast University, Nanjing 210096, China; xwbao@seu.edu.cn (X.B.); whfang@seu.edu.cn (W.F.); cmzhao@seu.edu.cn (C.Z.); 2 Purple Mountain Laboratories, Nanjing 211100, China

**Keywords:** MIMO, uplink precoding, limited feedback precoding, MMSE receivers, convolutional neural networks (CNNs), joint transceiver design

## Abstract

Multi-user multiple-input multiple-output (MU-MIMO) technology can significantly improve the spectral and energy efficiencies of wireless networks. In the uplink MU-MIMO systems, the optimal precoder design at the base station utilizes the Lagrange multipliers method and the centralized iterative algorithm to minimize the mean squared error (MSE) of all users under the power constraint. The precoding matrices need to be fed back to the user equipment to explore the potential benefits of the joint transceiver design. We propose a CNN-based compression network named PCQNet to minimize the feedback overhead. We first illustrate the effect of the trainable compression ratios and feedback bits on the MSE between the original precoding matrices and the recovered ones. We then evaluate the block error rates as the performance measure of the centralized implementation with an optimal minimum mean-squared error (MMSE) transceiver. Numerical results show that the proposed PCQNet achieves near-optimal performance compared with other quantized feedback schemes and significantly reduces the feedback overhead with negligible performance degradation.

## 1. Introduction

In recent years, the multi-user multiple-input multiple-output (MU-MIMO) technology has offered great advantages over conventional point-to-point MIMO systems due to its improvement on the spectral and the energy efficiencies [1,2]. Specifically, the base station (BS) of a MU-MIMO system communicates with a large number of user terminals in the same time-frequency resource by configuring numerous antennas. Furthermore, multiple antennas bring large improvements in throughput and radiated energy efficiency through focusing energy into ever smaller regions of space [3]. As a result, the MU-MIMO systems have become a fundamental and integral part of present and future generations of wireless networks. Digital beamforming and hybrid analog/digital beamforming are widely applied for inter-user interference reduction with the evolution and growth of 5G technical standards [4].

Nowadays, the joint optimization of the transceiver has attracted increasing research activities as an effective interference management technique for the uplink MU-MIMO systems [5]. Since the bottleneck of hardware cost and power consumption in the millimeter-wave mmWave Massive MIMO system will not appear in the uplink MU-MIMO scenarios, we adopt the digital precoding for its excellent performance in terms of sum rates. Some early works adopt the non-iterative [6,7] and iterative methods [8,9] to solve the highly non-convex problem of the joint transceiver optimization. The non-iterative precoding schemes are based on matrix decomposition, such as the singular value decomposition [6] and the QR decomposition [7], which cannot cope with the mismatch between the numbers of transmitting streams and the antennas. We note that the centralized iterative precoding scheme utilizing the method of Lagrange multipliers can solve the mismatch between the numbers of transmitting streams and the antennas. This iterative precoding scheme has the best end-to-end performance in the joint linear transceiver design but requires a certain feedback overhead [9]. In another aspect of studies, the high complexity nonlinear detector is unfeasible. Linear detectors such as the zero-forcing (ZF) detector and the minimum mean-squared error (MMSE) detector are widely applied for practical systems.

The online implementation of the centralized solution with the optimal MMSE receiver requires the necessary information feedback for the user equipment (UE) to perform real-time updating, whereas the offline one requires the feedback of the optimal precoding matrix. With the subsequent development of beyond fifth-generation (B5G) or sixth-generation (6G) technologies, the dimensions of the precoding matrices scales with the number of antennas, which gradually become rather large [10,11]. The increasing feedback overhead of precoding becomes a challenge for the high spectral efficiency, ultra-low latency, and high reliability requirements of future B5G and 6G wireless networks. Therefore, the design of new feedback architecture that lowers the feedback overhead and maintains high performance is crucial to unlocking the full potential of the uplink MU-MIMO.

Limited feedback of precoding techniques has been intensively investigated to reduce the feedback overhead in the uplink wireless communication systems [12,13,14], which mainly focuses on the design of a low-complexity codebook. An efficient precoding scheme is proposed in [12], where the optimal precoder is chosen from a finite codebook known to both the transmitter and the receiver. The application of the Lloyd-Max algorithm in [13] can be viewed as a vector quantization problem of codebook design. A three-dimensional MU-MIMO codebook is proposed in [14] which adopts the signal-to-noise ratio (SNR) maximization criterion to select the optimal codebook. The 3rd generation partnership project (3GPP) [15] provides a dedicated specification for the precoding matrix indexes (PMIs). Consequently, this protocol scheme has limited versatility, quantity, and accuracy performance. By contrast, the non-codebook-based centralized scheme has enormous throughput advantages compared to the codebook approaches but with relatively high cost of the feedback of precoding matrices.

Recently, as the success of deep learning reaches more fields, the neural-network-based auto-encoder has been recently applied to enhance the performance of MU-MIMO systems in [16,17,18]. It is worth noting that the auto-encoder is well suited to tackling the vector compression problem because of its robustness to the unstable wireless channel conditions. The deep neural network (DNN) in [19] takes the place of the conventional zero-forcing detection and offers near-optimal transmission quality with much less computational complexity than the optimal scheme. Motivated by the convolutional neural network (CNN)-based deep learning compression approaches of channel state information (CSI) [20,21,22], we propose a novel compression and quantization network architecture named PCQNet for the joint transceiver optimization. The proposed PCQNet compresses the high-dimensional precoding matrices to the low-dimensional vectors. Considering that only bitstreams can be transmitted in a practical digital system, we also introduce a quantization module to convert the floating-point vector into bitstreams. Our proposed PCQNet can flexibly adjust the compression ratios compared with the codebook scheme in [13]. These CNN-based methods considerably improve the compression performance of the precoding matrices. At the same time, the data-bearing bitstreams are directly produced during the offline training. Thus, the robustness of the network for practical deployment is effectively improved on the basis of the compression network in [19]. Moreover, we extend the CNN-based compression network in [20,21] to the precoding design of the uplink MU-MIMO scenarios.

Multiple works of the transceiver optimization are limited to single-user scenarios or the particular MU multiple-input single-output (MISO) systems [23,24]. We focus on the more general MU-MIMO scenarios considering both the interference of multiple users and the interference from multiple data streams of the same UE. We aim to design a trainable compression architecture for the offline implementation of the centralized precoding with the MMSE receiver. In summary, the major contributions are summarized as follows:

1. We propose a CNN-based architecture named PCQNet to produce the data-bearing bitstreams for each UE to recover the precoding matrices. It can achieve near-optimal performance and further reduce the feedback overhead compared with the existing 3GPP codebook scheme in certain scenarios.
2. We develop a general trainable compression and quantization framework for the precoding matrices in the uplink MU-MIMO systems. The proposed PCQNet architecture as well as the Lloyd-Max quantization scheme can flexibly adjust the feedback overhead by training an auto-encoder.
3. The precoding matrices with different compression ratios (CRs) are evaluated on the performance of the centralized implementation with an optimal MMSE transceiver. As far as we know, the effect of the feedback accuracy on the performance has not been investigated before. Specifically, we explore the trade-off between the block error rates (BLER) and the CRs of the precoding matrices.

The remainder of this article is organized as follows: Section 2 introduces the system model and the joint transceiver optimization. Section 3 describes the network architecture and the training strategy of the PCQNet. Three baseline methods are also presented to provide a benchmark for our proposed PCQNet. In Section 4, experimental evaluations and performance analysis are provided to demonstrate the efficiency of our trainable CNN-based PCQNet. Finally, the concluding statements are given in Section 5.

**Notations**: Symbols for matrices (vectors) are denoted by boldface upper (lower) case letters. R, C and N denote the real set, the complex set, and the positive integers, respectively. $C^{M\times N}$ denotes the M×N dimensional complex matrix space. ${\left(\cdot \right)}^{H}$, ${∥\cdot ∥}_{F}$, ${∥\cdot ∥}_{2}$, Tr(·), and E[·] denote the conjugate transpose, the Frobenius norm, the Euclidean norm, the trace operation, and the expectation, respectively. $I_{N}$ is the N×N identity matrix. $CN\left(\mu ,\sigma^{2}\right)\;\;$ is a complex Guassian vector with mean μ and variance $\sigma^{2}$.

## 2. System Model

In this section, we introduce a simple signal model of an uplink MU-MIMO system, the joint transceiver design and the channel models.

### 2.1. Uplink MU-MIMO System

**Figure 1:**
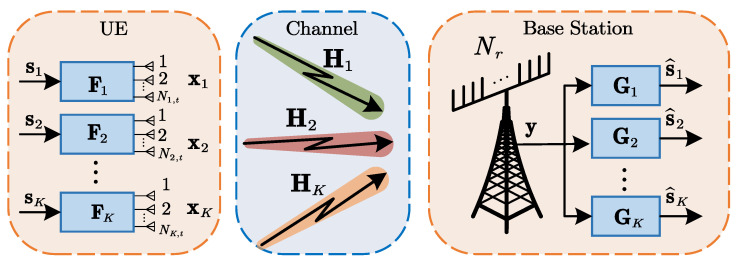
Block diagram of an uplink MU-MIMO wireless system with an $N_{r}$-antenna BS and *K* UEs each with $N_{k,t}$ antennas.

Without loss of generality, we consider the uplink MU-MIMO system consisting of one BS equipped with $N_{r}$ antennas and *K* UEs as depicted in Figure 1. For convenience, $K=\left\{1,\ldots ,K\right\}$ denotes the set of UEs. The *k*-th UE equipped with $N_{k,t}$ antennas transmits $N_{k,s}$ modulated data streams. We denote $N_{t}=\sum_{k=1}^{K}N_{k,t}$, $N_{s}=\sum_{k=1}^{K}N_{k,s}$ as the total numbers of transmit antennas and independent data streams of all UEs, respectively. For simplicity, we consider the case where $N_{k,t}$ and $N_{k,s}$ are constants. The channel matrix can be represented as $H≜\left[H_{1},\ldots ,H_{K}\right]\in C^{N_{r}\times N_{t}}$, where $H_{k}\in C^{N_{r}\times N_{k,t}}$ denotes the channel matrix from the *k*-th UE to the BS.

The BS firstly obtains the CSI of all UEs and calculates the optimal precoding matrices $F_{k}\in C^{N_{k,t}\times N_{k,s}}$ of the *k*-th (k∈K) UE which will be fed back to the UEs for the deployment of uplink data transmission. The received signal vector $y\in N_{r}$ at the BS can be represented as

(1) $$y=H_{k}F_{k}s_{k}+\sum_{i\neq k}H_{i}F_{i}s_{i}+n$$

where $s_{k}\in C^{N_{k,s}\times 1}$ represents the data symbol vector with a covariance matrix $\Phi_{s_{k}}=E\left[s_{k}s_{k}^{H}\right]=I_{N_{k,s}}$ and $n\in C^{N_{r}\times 1}$ is the received complex white Gaussian noise vector consisting of independent and identically distributed (i.i.d.) elements with the distribution $CN\left(0,\sigma^{2}I_{Nr}\right)$. The noise covariance matrix is $\Phi_{n}=\sigma^{2}I_{Nr}$.

The precoding and the linear detection are jointly optimized to obtain the best system performance. The recovered data symbol of the *k*-th UE can be represented by

(2) $${\hat{s}}_{k}=G_{k}y$$

where $G_{k}$ represents the detection matrix of the BS. The centralized implementation in [9] aims to jointly optimize the transceiver to eliminate the MU interference in the uplink system by minimizing the mean squared error (MSE) between the estimated symbols and the transmitted symbols. The MSE between the estimated symbols and the actual symbols for the *k*-th UE is given by

(3) $$\eta =\sum_{k=1}^{K}E\left[{\left({\hat{s}}_{k}-s_{k}\right)}^{H}\left({\hat{s}}_{k}-s_{k}\right)\right]$$

### 2.2. The Joint Precoding and Detection Design

In this paper, we consider the scheme in [9] which jointly designs the precoding and detection matrices to minimize the MSE between the estimated and the transmitted symbols. Specifically, we work on the overall system performance with the joint transceiver design which is subjected to the per user power constraint. The sum-MSE can be formulated as

(4) $$\begin{array}{cc} \min_{{\left\{F_{k},G_{k}\right\}}_{k=1,\ldots ,K}} & \eta \\ s.t.\;\;\;\;\;\;Tr\left\{F_{k}F_{k}^{H}\right\}⩽p_{k}, & \;\;k=1,\ldots ,K \end{array}$$

where the precoding matrix $F_{k}$ is subject to the per user power constraint $P_{k}=E\left\{{∥F_{k}s_{k}∥}^{2}\right\}=tr\left\{F_{k}\Phi_{sk}F_{k}^{H}\right\},\forall k\in K$. The joint minimization of MSE by iteratively updating the transceiver is carried out as follows

(5) $$G_{k}=F_{k}^{H}H_{k}^{H}{\left(\sum_{j=1}^{K}H_{j}F_{j}F_{j}^{H}H_{j}^{H}+\sigma^{2}I\right)}^{-1}\;$$

(6) $$F_{k}={\left(H_{k}^{H}\left(\sum_{i=1}^{K}G_{i}^{H}G_{i}\right)H_{k}+\lambda_{k}I\right)}^{-1}H_{k}^{H}G_{k}^{H},\;\;\forall k$$

where $\lambda_{k}$ is the Lagrange multiplier associated with the user power constraint. The detailed minimization process to obtain the optimal transceiver is described in Algorithm 1. The precoding matrix F is initialized with codebooks in the 3GPP protocol, which is simultaneously normalized to satisfy the power constraints. The Lagrangian formulation is utilized to solve the jointly convex optimization problem. The MMSE detection can optimally balance the multi-user interference and Gaussian noise compared with the ZF detector, Thus, we apply the MMSE detector in the proposed uplink scenario because of its practical implementation and better performance. The output-precoding matrix $F_{k}$ and detection matrix $G_{k}$ are utilized for precoding and MMSE detection at the *k*-th UE and the BS, respectively.
**Algorithm 1:** The centralized implementation with optimal MMSE precoding.
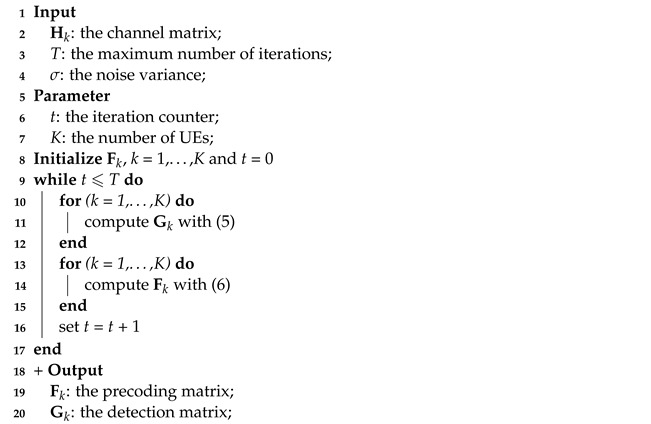


### 2.3. The Feedback Process of Precoding Matrices

**Figure 2:**
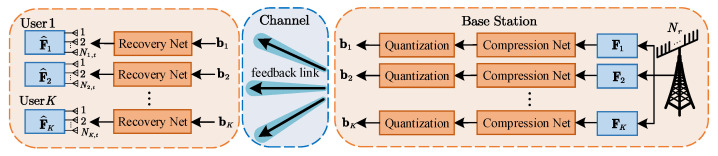
Block diagram of the feedback link for the precoding matrices over an uplink MU-MIMO System.

The full feedback of the precoding matrix imposes particularly high feedback overhead and storage requirements. We propose a CNN-based PCQNet scheme illustrated in Figure 2 to substantially decrease the feedback overhead. Specifically, the BS compresses the precoding matrices and then quantizes the compressed matrices to bitstreams for each UE. The UEs recover the precoding matrices utilizing an error-free transmission of the feedback bitstreams.

### 2.4. Channel Model

The channels are assumed to be frequency flat and known at the receiver side. We consider multiple channel models such as the i.i.d. Rayleigh fading channel and the more realistic NAIE channel provided by Huawei Corporation [25]. The channel matrix $h_{i,j}^{k}={\left[H_{k}\right]}_{i,j}$ represents the channel fading coefficient between the *j*-th transmit antenna of the *k*-th UE and the *i*-th received antenna of the BS. The i.i.d. Rayleigh channel matrix H consists of independent and identically distributed $\left(i.i.d.\right)$$\mathrm{CN}\left(0,1\right)$ elements. The NAIE MIMO channels are taken from the CDLB300_20UE_4T32R dataset provided by the iMaster NAIE platform, which is a channel environment measured in practical scenarios.

The parameters of the NAIE dataset are listed in Table 1. The dimension of the dataset matrices is $\left[L,K,N_{k,t},N_{r},N_{f}\right]=\left[500,20,4,32,96\right]$, which represents the number of data frames, the number of UEs, the number of antennas for UE, the number of antennas for BS, and the number of carriers, respectively. When the testing and training datasets are generated for the NAIE channel, they are randomly picked from the 500 matrix elements and then normalized to satisfy the power constraints. If the number of frames is less than 500, the data frames are randomly selected. Otherwise, the datasets will be reused when the number of data frames is larger than the dimension of the dataset L=500.

## 3. The Proposed PCQNet

### 3.1. Network Architecture

**Figure 3:**
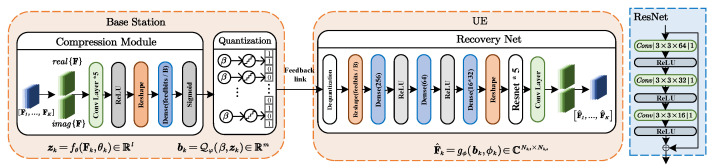
The encoder and decoder architecture of CNN-based PCQNet.

The PCQNet consists of the encoder network and the recovery network as illustrated in Figure 3. The encoder network is made up of a trainable compression module and a non-trainable quantization module. The quantization is accomplished during the offline training, directly feeding back the codeword to the UEs through the data-bearing bitstreams. The recovery network mainly consists of a dequantizer and the ResNet in [26].

Considering that the CNNs can efficiently manage the memory requirements on-device and achieve better memory usage than the DNNs, we apply the CNNs with growing popularity for the compression networks. We firstly concatenate the real and the imaginary parts of the precoding matrices $F_{k}$, k∈K, where the dimension transformation can be represented by $C^{N_{k,t}\times N_{k,s}}\mapsto R^{2\times N_{k,t}\times N_{k,s}}$. The input of the first convolutional layer is the real and imaginary parts of the precoding matrices generated at the BS. The compression module consists of five CNN layers which create a filter kernel that is convolved with the input layer to produce an output tensor. The CNN layers are parameterized by F×F×K|S, where F and K denote the filter size and the number of filters, respectively. S represents the downsampling strides in the convolution layer at the encoder and the upsampling strides in the transposed convolution layer at the decoder. The hyperparameters of five CNN layers in the compression module are: 3×3×32|1, 3×3×32|1, 3×3×32|1, 3×3×32|1, 3×3×16|1, respectively. The linear unit (ReLU) activation function is inserted after each CNN layer. The output is the compressed vector $z_{k}\in R^{l}$. The compression function $f_{\theta }:C^{N_{k,t}\times N_{k,s}}\mapsto R^{l}$ can be represented as

(7) $$z_{k}=f_{\theta }\left(F_{k},\theta_{k}\right)\in R^{l},k=1,\ldots ,K$$

where $\theta_{k}$ is the same parameter for each user, *l* is the dimension of the compressed output. We use a uniform quantization module with the quantization factor β. The vector $z_{k}$ is quantized into an *m*-dimensional binary vector $b_{k}$ for the feedback transmission of the *k*-th UE.

(8) $$b_{k}=Q_{\varphi }\left(\beta ,z_{k}\right)\in R^{m}$$

where *m* represents the feedback overhead. For each UE, the CR of the PCQNet can be defined as

(9) $$\mathrm{CR}=\frac{m}{2\beta \times N_{k,t}\times N_{k,s}}$$

The CR and the number of quantization bits β jointly determine the feedback overhead *m* which influences the normalized MSE (NMSE) between the recovered precoding matrices and the original ones. A smaller value of *m* reflects lower feedback overhead of the precoding matrices. The binary vector $b_{k}\in R^{m}$ is fed to the UE for the recovery of the precoding matrices. An error-free channel is assumed when transmitting the encoded vector $b_{k}$ from the BS to the *k*-th UE.

The decoder network at the *k*-th UE outputs the restored complex-valued precoding matrix ${\hat{F}}_{k}$ from the feedback bitstream $b_{k}$. The reconstruction of the precoding matrix can be functioned with $g_{\phi }:R^{m}\mapsto C^{N_{k,t}\times N_{k,s}}$

(10) $${\hat{F}}_{k}=g_{\phi }\left(b_{k},\phi_{k}\right)\in C^{N_{k,t}\times N_{k,s}},k=1,\ldots ,K$$

where $\phi_{k}$ represents the parameter sets of the decompression module. The feedback bitstream $b_{k}$ is reshaped to the dimension of m/β. The decompression module retrieves the real and the imaginary parts from three fully connected (FC) layers and five ResNet layers. The ResNet applies shortcut connections that directly pass data flow to later layers to avoid the vanishing of the gradient caused by multiple stacked non-linear transformations. Each of the FC layer is followed by a ReLU activation and the hyperparameters of three ResNets in decoder are: 3×3×64|1, 3×3×32|1, and 3×3×16|1, respectively.

### 3.2. The Training Strategy of PCQNet

In the offline stage, we compute the precoding matrices in advance by the aforementioned Algorithm 1 and generate the training, testing, and evaluation datasets. In the online stage, we can directly obtain the low dimensional feedback bitstreams with the well-trained neural network. The gradient of the quantization module is treated as a constant to make the network differentiable, and for this reason the encoder and the recovery network can be trained end-to-end. We jointly optimized the encoder and the decoder modules with back-propagation and gradients can pass through the quantization layer during back-propagation.

We formulate the feedback of the precoding matrices into a reconstruction problem by ${\hat{F}}_{k}=g_{\phi }\left(Q_{\varphi }\left(\beta ,f_{\theta }\left(F_{k},\theta_{k}\right)\right),\phi_{k}\right)$. The auto-encoder is optimized by updating the network parameters $\theta_{k}$ and $\phi_{k}$, which can be applied for all UEs. The loss function is the NMSE, which quantifies the difference between the recovered precoding matrices and the original ones with

(11) $$\mathrm{NMSE}=\frac{E\left[{∥\hat{F}-F∥}_{F}^{2}\right]}{E\left[{∥F∥}_{F}^{2}\right]}$$

The PCQNet is trained and evaluated on an Nvidia GeForce 3090 platform. We use the Adam [27] optimizer with a batch size of 32 and the training process stops early with a patience of eight epochs, where the maximum number of training epochs is 1000. We apply the adaptive learning rate schedule with a factor of 0.8. If the loss does not improve for four epochs in a row, the learning rate is reduced.

### 3.3. Testing of PCQNet

#### 3.3.1. Baseline1: The Protocol Codebook-Based Precoding Scheme

The 3GPP protocol in [15] contains a set of PMIs $F_{\mathrm{codebook}}^{\left(i\right)},i\in N$ with corresponding configurations of streams and antennas. The BS firstly calculates the precoding matrix with Algorithm 1 and then feeds back the binary index. The optimal codebook index $i^{\left(opt\right)}$ is acquired with the minimum Euclidean distance by searching the predefined codebooks

(12) $$i^{\left(opt\right)}=\arg\min_{i}{∥F-F_{\mathrm{codebook}}^{\left(i\right)}∥}_{F}^{2}$$

where *i* represents the index of the standard precoding matrix defined in the 3GPP protocol supporting limited scenarios (e.g., $N_{k,t}=4$, $N_{k,s}=2$, $i^{\left(opt\right)}\in \left[0,21\right]$ ). The number of feedback bits for the protocol codebook scheme under the scenarios with 22 indexes is 5 ($m=\lceil \log_{2}22\rceil =5$).

The protocol codebook-based precoding scheme is labeled as **3GPP codebook** in our simulation. Since this feedback method of the vector quantization only needs to search the optimal index, it greatly reduces the feedback overhead. However, the scope of the codebook scheme is limited and the precoding matrices retrieved from the codebook indexes inevitably have certain quantization error.

#### 3.3.2. Baseline2: The Lloyd-Max Quantization Scheme

We apply the Lloyd-Max quantizer to reduce the dimension of the feedback matrices which is labeled as LloydMax in our simulation. An optimized Lloyd-Max quantizer minimizes the mean square quantization error (distortion) as much as possible. The Lloyd-Max quantization scheme stores the designed codebooks and partitions at the UEs and the BS, thus the BS only needs to transmit the indexes to specify the precoding matrices.

In the offline stage, we first acquire the datasets generated by Algorithm 1. The empirical probability distribution function of the real and the imaginary parts of the precoding matrices is obtained for the design of the Lloyd-Max quantizer in [13,28,29]. Then, we develop a Lloyd-Max-based quantizer under different SNRs and channel models. The codebooks and partitions are specifically optimized by the Lloyd-Max algorithm in [30,31].

In the online stage, the UEs can readily recover the precoding matrices utilizing the received indexes with the prestored Lloyd-Max quantization partitions and codebooks. Each element in the precoding matrices has to be quantized individually, thus the minimum number of feedback bits is $N_{k,t}\times N_{k,s}\times 2$ for each precoding matrix $F_{k}\in C^{N_{k,t}\times N_{k,s}}$. (e.g., $N_{k,t}=4,N_{k,s}=2,\beta =1,m=16$). It is necessary to further reduce the feedback overhead and design a more SNR-adaptive feedback scheme to combat channel variations.

#### 3.3.3. Baseline3: The Ideal Feedback Scheme

We consider the optimal scheme labeled as w/o compression which directly feeds back the precoding matrices without compression.

## 4. Experimental Evaluations

In this section, we evaluate the NMSE of the precoding matrices with different CRs and the influence of the feedback accuracy on the BLER performance. The comprehensive performance comparisons of the uplink MU-MIMO system with different number of UEs, different modulation orders, and different channel models are provided. The uplink MU-MIMO system parameters and the coefficients of the channel coding are listed in Table 2. The SNR is generally defined as the ratio of the signal power to the noise power at the receiver

(13) $$\mathrm{SNR}=\frac{E\left[{∥\mathrm{HFs}∥}_{2}^{2}\right]}{E\left[{∥n∥}_{2}^{2}\right]}=\frac{N_{t}K}{\sigma^{2}}$$

### 4.1. Data Generation

We firstly generate *L* channel realizations $\left\{H^{1},\ldots ,H^{L}\right\}\in C^{L\times N_{r}\times N_{t}}$ for the i.i.d. Rayleigh channel or randomly sample *L* data frames from the iMaster NAIE platform [25] for the NAIE channel. Then, we calculate the noise variance and normalize the channel matrix. The precoding matrices $F^{l}≜\left[F_{1},\ldots ,F_{K}\right]\in C^{N_{t}\times N_{s}},l=1,\ldots ,L$ are generated by the channel matrices $H^{l}=\left[H_{1},\ldots ,H_{K}\right],l=1,\ldots ,L$ utilizing the Algorithm 1. Lastly, we sample *L* training data $F=\left\{\left(H^{1},F^{1}\right),\ldots \left(H^{L},F^{L}\right)\right\}$.

At the training and evaluation stage for the networks, the precoding matrix $F_{k}\in C^{N_{k,t}\times N_{k,s}}$ of the *k*-th UE is randomly picked from the set of $[F^{1},\ldots ,F^{L}],l=1,\ldots ,L$. The number of frames of the training, validation, and testing data for our proposed PCQNet are $L_{1}$ = 100,000, $L_{2}$ = 20,000, $L_{3}$ = 10,000, respectively.

### 4.2. Simulation Results and Analysis with the i.i.d. Rayleigh Channel

#### 4.2.1. The NMSE Performance

The NMSE performance between the recovered and the original precoding matrices utilizing the **LloydMax** scheme is depicted in Table 3. The codebooks are specially optimized over statistical datasets with various SNRs and different quantization bits. Since these codebook-based schemes are inevitably limited by the quantization error, better NMSE performance of the LloydMax scheme comes at the expense of the increased feedback overhead. Although remarkable NMSE performance can be obtained when the evaluating SNRs (i.e., ${\mathrm{SNR}}_{eval}$) match with the designing SNRs (i.e., ${\mathrm{SNR}}_{design}$) under the same quantization bits, the performance of the codebook-based scheme is not satisfactory when ${\mathrm{SNR}}_{eval}$ mismatch with the ${\mathrm{SNR}}_{design}$ over the i.i.d. Rayleigh channel. Thus, it needs to store multiple codebooks to combat channel variations under different channel SNRs.

**Figure 4:**
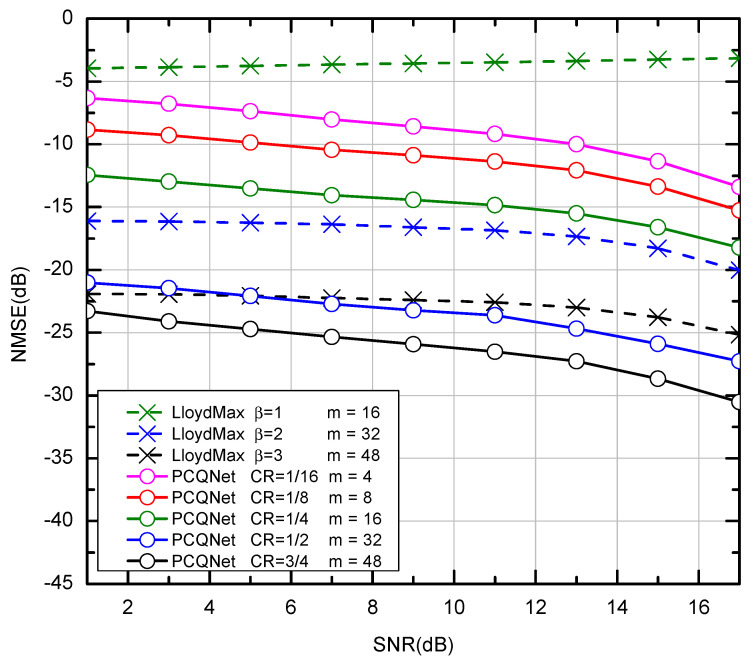
The NMSE performance of comparison between the Lloyd-Max scheme and the CNN-based PCQNet scheme over the i.i.d. channel. ( The x-coordinate represents the SNRs for the generation of the testing dataset, which are not directly correlated with the NMSE performance).

The NMSE performance of the LloydMax scheme and the PCQNet is not correlated with SNRs. Only the generation of different test datasets is related to SNRs and the testing of NMSE performance is not necessarily related to the value of the SNRs. We provide a guideline for subsequent research of BLER performance via the visualization of NMSE performance in Figure 4.

The codebook-based LloydMax scheme is sensitive to the number of bits, the SNRs, and the realistic channel distribution. The NMSE will significantly drop when the ${\mathrm{SNR}}_{eval}$ mismatches with the ${\mathrm{SNR}}_{design}$. On the contrary, the CNN-based compression scheme is more SNR-adaptive and we set ${\mathrm{SNR}}_{design}$ = 0 dB. The CRs of the PCQNet are set to 1/16, 1/8, 1/4, and 1/2 for m = 4, 8, 16, 32, respectively. The PCQNet can achieve better NMSE performance than the LloydMax scheme under the same feedback bits (e.g., m=16,32,48) as shown in Figure 4. The green dotted line with low quantization factor β=1 is almost a straight line over the i.i.d. channel. Despite the different input precoding matrices under different SNRs, the NMSE of the recovered matrices is equally poor. Because the quantization error is so large that the precoding matrices cannot be correctly recovered.

#### 4.2.2. The BLER Performance

**Figure 5:**
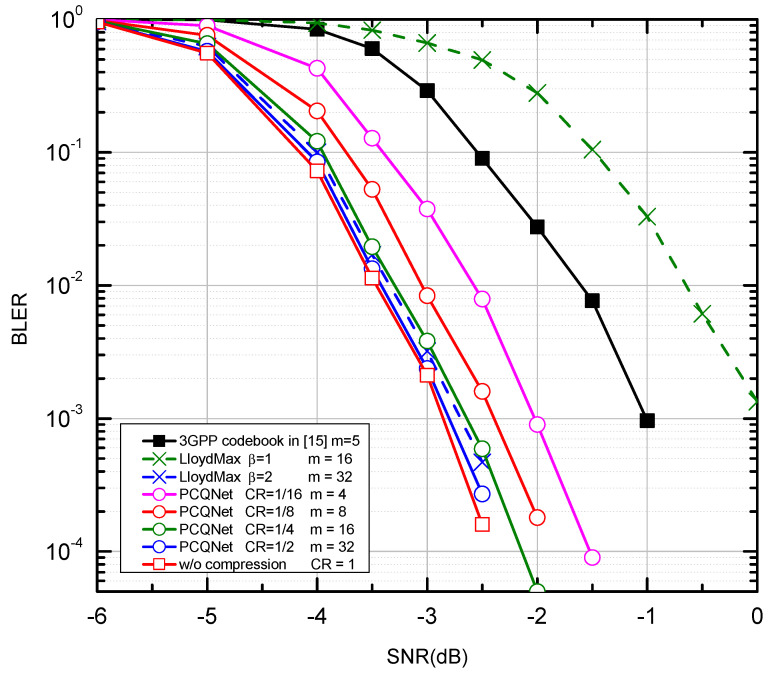
The BLER performance comparison over the i.i.d. Rayleigh channel with QPSK and K = 4.

**Figure 6:**
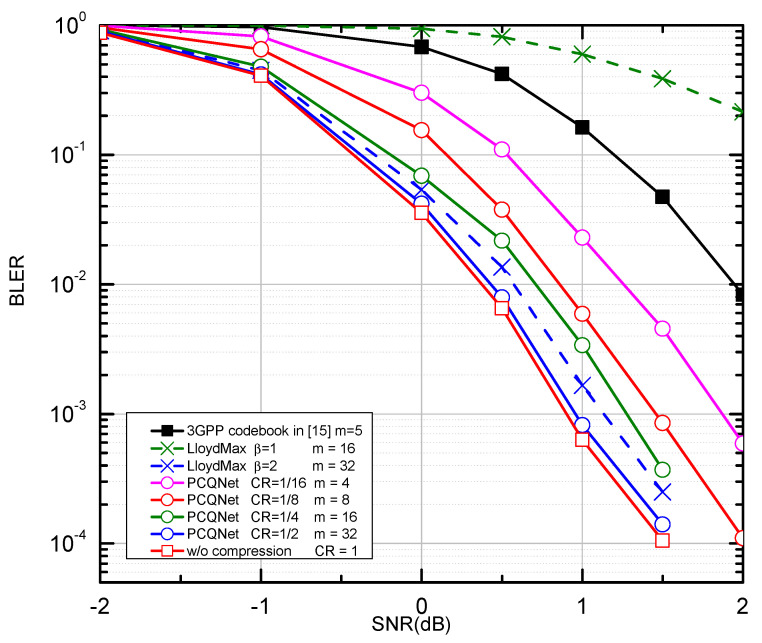
The BLER performance comparison over the i.i.d. Rayleigh channel with QPSK and K = 8.

We compare the PCQNet with the aforementioned baselines as depicted in Figure 5 and Figure 6. The comparisons of feedback overhead between the PCQNet and three baselines (i.e., the 3GPP codebook scheme, the w/o compression scheme, and the LloydMax scheme) are provided in Table 4. The performance upper bound is the ideal centralized iterative scheme w/o compression which is not appropriate for practical transmission. The protocol codebook-based precoding scheme is tailored for a specific number of users or transmit antennas. To provide a benchmark for our proposed method, we consider the precoding matrices $F_{k}\in C^{4\times 2},k\in \left\{1,2,3,4\right\}$ which is to be fed back to the *k*-th UE. The number of the feedback bits for the 3GPP codebook scheme is m=5. The LloydMax scheme separately quantizes the real and the imaginary parts of the precoding matrices, which respectively takes at least 16 bits and 32 bits to quantize a $R^{2\times 4\times 2}$ matrix with β = 1 and 2 (m=16×β = 16, 32).

The proposed PCQNet can dramatically decrease the feedback overhead and exhibit a slight BLER degradation with the further reduction of feedback bits (i.e., m=4,8). Note that the change of the CRs can be achieved by adjusting the number of feedback bits. Near-optimal performance can be derived when the number of the feedback bits is beyond 16, where the feedback overhead can be further reduced. When m=4, the PCQNet significantly enhances the BLER performance of the 3GPP codebook scheme with m=5. Naturally, as we increase the number of feedback bits to 32, the performance of the PCQNet scheme as well as the LloydMax scheme will approach that of the ideal w/o compression scheme.

### 4.3. Simulation Results and Analysis with NAIE Channel

We provide more simulation tests to evaluate the performance of the proposed scheme with various numbers of UEs and higher modulation orders (e.g., 16-QAM) as well as the NAIE channel in practical scenarios provided by the iMaster NAIE platform [25].

#### 4.3.1. The NMSE Performance

**Figure 7:**
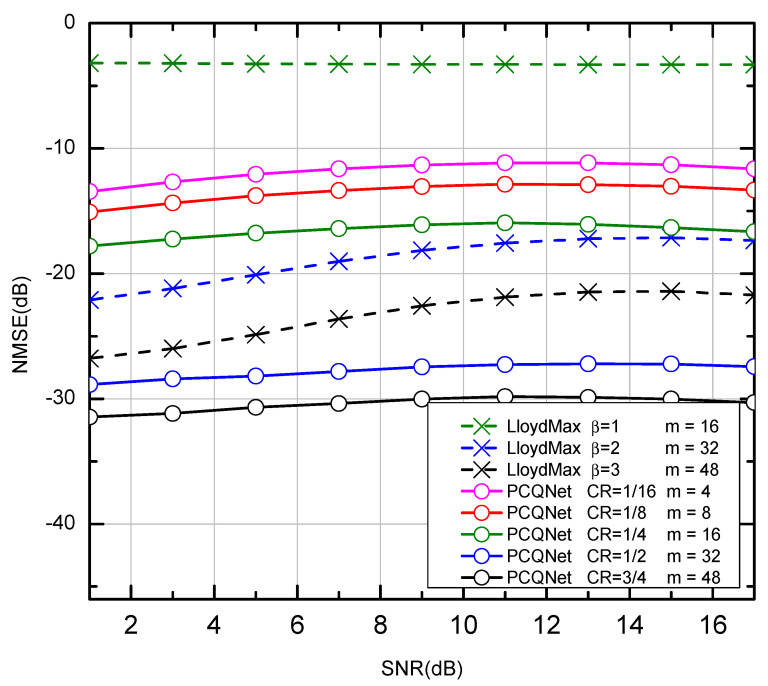
The NMSE performance of comparison between the Lloyd-Max scheme and the CNN-based PCQNet scheme over the NAIE channel. (The x-coordinates represent the SNRs for the generation of the testing dataset, which are not directly correlated with the NMSE performance).

The LloydMax scheme has to design multiple codebooks which are optimized for specific channel conditions. The best NMSE performance can be achieved when the ${\mathrm{SNR}}_{eval}$ is equal to the ${\mathrm{SNR}}_{design}$ in Table 5. The PCQNet has superior NMSE performance than the LloydMax scheme under the same CRs as depicted in Figure 7. The PCQNet is tested with the fixed training SNR value ${\mathrm{SNR}}_{design}$ = 0 dB while the LloydMax scheme is evaluated with ${\mathrm{SNR}}_{eval}$ = ${\mathrm{SNR}}_{design}$.

#### 4.3.2. The BLER Performance of the NAIE Channel

**Figure 8:**
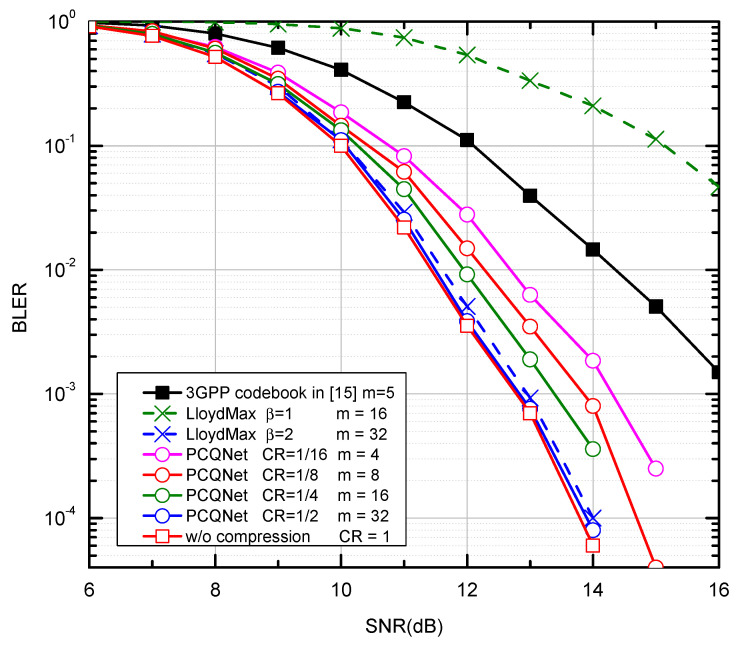
The BLER performance comparison over the NAIE Channel with 16-QAM and K = 6.

**Figure 9:**
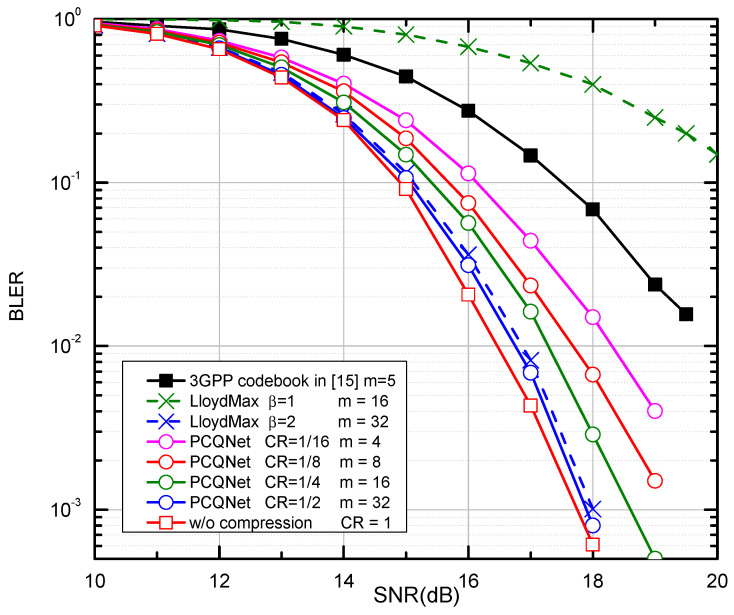
The BLER performance comparison over the NAIE Channel with 16-QAM and K = 8.

Compared with the w/o compression scheme which fully attains the precoding matrix, the CNN-based PCQNet can enhance the recovery quality of the precoding matrix with adaptive feedback overhead and obtain near optimal reconstruction performance. Similar performance can be seen over the NAIE channel in Figure 8 and Figure 9. The CNN-based PCQNet scheme performs close to the ideal w/o compression scheme when CR=1/2. Moreover, there is a slight performance penalty when CR=1/16 which is still superior to the 3GPP codebook. With the deployment of the pre-trained PCQNet, the feedback overhead is substantially reduced while the performance degradation is acceptable. The PCQNet scheme has better BLER performance than the LloydMax scheme under the same CRs.

The PCQNet achieves the near-optimal BLER performance when the NMSE of the recovered precoding matrix is lower than −20 dB. The transmission tends to stop if the NMSE of the recovered precoding matrices is worse than the threshold of −5 dB. We also observe that, when the NMSE performance exceeds a certain threshold (e.g., −20 dB), the overall BLER performance is quite close to the ideal w/o compression scheme. From the results, we can see that it is a reasonable compromise to set the CR to 1/4 for the compression of precoding matrices.

The provided numerical results show that our proposed PCQNet achieves a better trade-off between the feedback overhead and the BLER performance over the i.i.d. Rayleigh channel and the NAIE channel. This CNN-based compression scheme significantly enhances the BLER performance compared with the 3GPP codebook scheme and the LloydMax scheme under the same CRs.

## 5. Conclusions

The proposed PCQNet has achieved considerable gains in BLER performance compared with the protocol codebook-based precoding scheme and the Lloyd-Max quantization scheme under the same CRs. The adaptability of trainable PCQNet architecture to different channel bandwidths is more competitive than the Lloyd-Max quantization scheme in bandwidth-limited scenarios. The PCQNet also provides better resilience to the mismatch between the trained SNRs and tested SNRs than the Lloyd-Max quantization scheme due to channel variations. Our experiments demonstrate that the application of the CNN-based PCQNet greatly improves the adaptability and the generality of the precoding matrix feedback in the uplink MU-MIMO systems. Importantly, it preserves only a slight degradation of BLER performance with high compression rate of the precoding matrix, making the compression architecture more attractive for the deployment of practical systems.

## Figures and Tables

**Table 1:** Description of parameters of the NAIE dataset for uplink scenarios.

Parameter	Description	Settings
L	The number of data frames	500
*K*	The number of UEs	20
$N_{k,t}$	The number of antennas of each UE	4
$N_{r}$	The number of antennas of BS	32
$N_{f}$	The number of subcarriers	96

**Table 2:** List of simulation parameters.

Parameter	Description	Settings
*K*	The number of UEs	4,6,8
$N_{k,t}$	The number of antennas of each UE	4
$N_{k,s}$	The number of data streams of each UE	2
$N_{t}$	The total number of transmit antennas	16, 24, 32
$N_{s}$	The total number of independent data streams	8, 12, 16
$N_{r}$	The number of BS antennas	32
M	The number of bits of per modulated symbol	2, 4
N,Rate	The length and code rate of the low density parity check code	384, 1/2
$\beta_{1}$	The quantization bits of the Lloyd-Max scheme	2,3
$\beta_{2}$	The quantization bits of the PCQNet	4
$m_{1}$	The numbers of feedback Bits of the Lloyd-Max scheme	16, 32, 48
$m_{2}$	The numbers of feedback Bits of the PCQNet scheme	4, 8, 16, 32, 48
CR	The CRs of the PCQNet scheme	1/16, 1/4, 1/2

**Table 3:** The NMSE performance of the Lloyd-Max quantization scheme with different quantization factors over the i.i.d. channel.

Quantization Factor		β = 2 (*m* = 32)			β = 3 (*m* = 48)		
**NMSE (dB)**	**SNR** ${}_{\mathrm{design}}$ **(dB)**	**1**	**9**	**17**	**1**	**9**	**17**
**SNR** ${}_{\mathrm{eval}}$ **(dB)**							
	1	−16.16	−15.23	−11.98	−21.93	−21.14	−17.61
	9	−15.93	−16.60	−16.23	−21.67	−22.37	−20.20
	17	−15.37	−17.79	−20.08	−20.07	−23.01	−25.27

**Table 4:** The comparison of feedback overhead between the PCQNet and three baselines.

Methods	The Feedback Overhead (Number of Feedback Bits)
The 3GPP protocol codebook	5
The LloydMax scheme	16, 32
* The PCQNet scheme	4, 8, 16, 32
w/o compression (ideal)	a complex matrix with size (4, 2)

* Our proposed PCQNet. System Paramters: Number of UEs = 4, *N*_*k*,*t*_ = 4, *N*_*k*,*s*_ = 2, *N*_*r*_ = 32.

**Table 5:** The NMSE performance of Lloyd-Max quantization scheme with different quantization factors over the NAIE channel.

Quantization Factor		β = 2 (*m* = 32)			β = 3 (*m* = 48)		
**NMSE (dB)**	**SNR** ${}_{\mathrm{design}}$ **(dB)**	**1**	**9**	**17**	**1**	**9**	**17**
**SNR** ${}_{\mathrm{eval}}$ **(dB)**							
	1	−22.10	−21.00	−21.42	−26.78	−26.06	−26.44
	9	−18.14	−18.95	−18.78	−22.57	−24.29	−23.88
	17	−17.34	−18.44	−19.19	−21.71	−23.84	−24.32

## Data Availability

Not applicable.
